# A systematic literature review of economic evaluation studies of interventions impacting antimicrobial resistance

**DOI:** 10.1186/s13756-023-01265-5

**Published:** 2023-07-13

**Authors:** Chris Painter, Dian Faradiba, Kinanti Khansa Chavarina, Ella Nanda Sari, Yot Teerawattananon, Kristina Aluzaite, Aparna Ananthakrishnan

**Affiliations:** 1 Health Intervention and Technology Assessment Program (HITAP), Ministry of Public Health, Nonthaburi, Thailand; 2 Saw Swee Hock School of Public Health, National University of Singapore, Singapore, Singapore; 3 Centre for Tropical Medicine and Global Health, Nuffield Department of Medicine, University of Oxford, Oxford, UK; 4 National University of Singapore, Singapore, Singapore; 5 Centre for Health Economics, University of York, York, UK

**Keywords:** AMR, Cost-effectiveness, Economic evaluation, Antibiotic resistance, Cost–benefit analysis

## Abstract

**Background:**

Antimicrobial resistance (AMR) is accelerated by widespread and inappropriate use of antimicrobials. Many countries, including those in low- and middle- income contexts, have started implementing interventions to tackle AMR. However, for many interventions there is little or no economic evidence with respect to their cost-effectiveness. To help better understand the scale of this evidence gap, we conducted a systematic literature review to provide a comprehensive summary on the value for money of different interventions affecting AMR.

**Methods:**

A systematic literature review was conducted of economic evaluations on interventions addressing AMR. a narrative synthesis of findings was produced. Systematic searches for relevant studies were performed across relevant databases and grey literature sources such as unpublished studies, reports, and other relevant documents. All identified economic evaluation studies were included provided that they reported an economic outcome and stated that the analysed intervention aimed to affect AMR or antimicrobial use in the abstract. Studies that reported clinical endpoints alone were excluded. Selection for final inclusion and data extraction was performed by two independent reviewers. A quality assessment of the evidence used in the included studies was also conducted.

**Results:**

28,597 articles were screened and 35 articles were identified that satisfied the inclusion criteria. The review attempted to answer the following questions: (1) What interventions to address AMR have been the subject of an economic evaluation? (2) In what types of setting (e.g. high-income, low-income, regions etc.) have these economic evaluations been focused? (3) Which interventions have been estimated to be cost-effective, and has this result been replicated in other settings/contexts? (4) What economic evaluation methods or techniques have been used to evaluate these interventions? (5) What kind and quality of data has been used in conducting economic evaluations for these interventions?

**Discussion:**

The review is one of the first of its kind, and the most recent, to systematically review the literature on the cost-effectiveness of AMR interventions. This review addresses an important evidence gap in the economics of AMR and can assist AMR researchers’ understanding of the state of the economic evaluation literature, and therefore inform future research.

*Systematic review registration* PROSPERO (CRD42020190310).

**Supplementary Information:**

The online version contains supplementary material available at 10.1186/s13756-023-01265-5.

## Introduction

The World Health Organization (WHO) listed antimicrobial resistance (AMR) as one of the top ten threats to global health in 2019, with estimates that AMR could cause 10 million deaths per year by 2050 [1, 2]. AMR occurs when pathogens (bacteria, viruses, fungi and parasites) develop a resistance or tolerance to the medicines that are used to combat these microorganisms, resulting in treatments becoming less effective or ineffective [3]. The COVID-19 pandemic illustrated how quickly the spread of new pathogens with no effective treatment can take place, and how devastating the effects can be. AMR is already thought to be responsible for millions of deaths each year [4], and although AMR is a naturally occurring phenomenon, the rate at which it occurs is impacted by exposure pathogens to antimicrobial agents and their selective pressure [5]. There are numerous documented cases of pathogens developing AMR, with some of the pathogens responsible for the most deaths globally including methicillin-resistant *Staphylococcus aureus* (MRSA), drug-resistant tuberculosis and drug-resistant *Escherichia coli* [4, 6].

AMR has increased in low-, middle- and high-income countries around the world in recent years and this pattern is expected to continue [7–9]. Klein et al. [10] conducted a trend analysis on antibiotic consumption between 2005 and 2015 in 76 countries. The results indicate that between this time period, antibiotic consumption rose globally by 65% (measured by defined daily doses [DDD], a standard drug intake metric), primarily driven by increases in consumption in low-and middle-income countries (LMICs); estimates suggest a 77% increase in antibiotic consumption rate per 1,000 inhabitants in these regions. In many cases, antibiotics are overused or misused (e.g. the use of antibiotics for common viral infections like the flu in humans and as growth promoters in farm animals) [11–13]. Though antibiotic use is rising in LMICs, lack of access to appropriate and effective antimicrobials is a continuing problem in these settings, due to issues with affordability, supply chains and substandard and falsified medicines, amongst others [14, 15].

AMR has been recognised as a One Health issue by key international organisations [16], interventions to reduce AMR should consider its multisectoral nature, recognising the links between antimicrobial use in humans, animals and the environment [17]. The WHO’s 2015 Global Action Plan on AMR identified several key methods for reducing AMR as a threat, examples of which included: optimisation of the use of antimicrobials in both human and animal health; reducing infections, through effective sanitation, hygiene and other infection prevention measures; sustainable investment in the development of new antimicrobials, diagnostic tools and other interventions [18].

Health technology assessment (HTA) is a multidisciplinary process that uses explicit methods to determine the value of health technologies or interventions at different points throughout the life cycle of the technology or intervention [19]. HTA often involves a health economic evaluation component which can produce comparative estimates of the cost-effectiveness of health interventions. In other words, economic evaluations can aid policymakers to understand whether certain interventions offer better value-for-money compared to other options. The Global Action Plan on AMR emphasised the need to promote more economic evidence-based use of interventions in development of a financial case for investment in AMR diagnostics and treatments [18]. However, some AMR interventions or initiatives have little or no evidence concerning their relative costs and benefits; such as educational and awareness raising interventions, or animal and environmental interventions. This is an issue of significant importance, particularly in more resource-constrained settings that need to use their budgets efficiently and where the burdens of AMR are rapidly rising [20].

The most recent systematic review on the cost-effectiveness of measures to contain the occurrence of AMR, looking at any type of intervention, dates back to 2002 [21], the study demonstrated that there was limited economic evidence available for AMR interventions. Since this review, research in the area has grown, although systematic reviews of evidence have focussed solely on the impact of antimicrobial stewardship programmes (ASPs) in hospitals, or the economic burden of AMR [22, 23].

### Objectives

A broad systematic review was conducted with the aim to summarise and detail data from economic evaluations regarding the value-for-money of interventions impacting AMR as a step towards optimising resource use. Specifically, this review attempted to answer the following questions:

- *Objective 1* What interventions to address AMR have been the subject of an economic evaluation?
- *Objective 2* In what types of setting (e.g. high-income, low-income, regions etc.) have these economic evaluations been focused?
- *Objective 3* Which interventions have been estimated to be cost-effective, and has this result been replicated in other settings/contexts?
- *Objective 4* What economic evaluation methods or techniques have been used to evaluate these interventions?
- *Objective 5* What kind of data has been used in conducting economic evaluations for these interventions? What is the quality of this data?

## Materials and methods

### Search strategy and study selection

Search strategies were designed for accessing MEDLINE (Ovid), EMBASE (embase.com), Cochrane Library, Web of Science, Tufts Cost Effectiveness Analysis (CEA) Registry and Global Health (GH) CEA Registry, Centre for Reviews and Dissemination’s National Health Service Economic Evaluation Database (NHS-EED). Grey literature searches were also conducted, including using international conference databases for the following conferences: Health Technology Assessment International (HTAi), International Society of Pharmacoeconomics and Outcomes Research (ISPOR), International Health Economics Association (iHEA). Websites and databases from HTA or regulatory agencies such as the National Institute for Health and Care Excellence (NICE) or the Canadian Agency for Drugs and Technologies in Health (CADTH) were not searched. Hand searches of the bibliographies of any included studies were performed to identify any overlooked articles of relevance.

Both trial-based and model-based economic evaluations published in the English language and from the year 2000 to 2021 were included, to capture studies that wouldn’t have previously been identified in the review by Wilton et al. [21]. Any category of full economic evaluation was included, which did not include budget impact analyses. Studies on interventions to reduce AMR which report only clinical endpoints and do not investigate any economic outcomes were excluded from the review. Reviews, editorials, commentaries, and methodological articles were excluded. A detailed description of the search strategy can be found in the associated publication for the systematic review protocol [24].

Data were exported to Covidence based on the inclusion and exclusion criteria of this review. The eligibility of the studies for review was assessed subjectively, and uncertainties were resolved in discussion amongst the reviewers. The inclusion of relevant studies was conducted with a two-step process: (1) Two reviewers independently screened titles and abstracts of all articles initially retrieved. (2) Full text screening of selected systematic reviews was conducted by two independent reviewers.

### Population, interventions and outcomes

The populations considered included humans, animals, and the environment, consistent with the One Health approach of addressing AMR [11]. To be included in the review, the abstract of the article must have stated that a considered intervention in the evaluation would have an impact on AMR in some way, for example, through controlling the spread of resistant microbials, eradicating resistant microbials, or reducing antimicrobial use. There was no limitation on the type of intervention that could be included in the review.

The types of outcome measures that this review recorded included any cost–benefit measurement such as incremental cost-effectiveness ratio (ICER), incremental cost per quality-adjusted life year (QALY), incremental cost per disability-adjusted life year (DALY), incremental cost–benefit ratio, net monetary benefit (NMB), incremental net benefit (INB), net health benefit (NHB), costs avoided, net costs, cost-consequence measures and budget impact. Our review also included outcomes that are specific to the AMR context, such as incremental cost per resistant infection avoided. Furthermore, this review analysed the types of settings (countries or regions, country income-levels, farms, pharmacies or hospitals, types of hospitals (primary, community or tertiary), that these analyses were focussed on.

### Data analysis and synthesis

A standardised template was created to facilitate the extraction of data from the included articles. Two researchers assisted in extracting the data of included studies. One researcher extracted the data and another researcher was responsible for checking the accuracy of the extracted data. In the case of disagreements, a third researcher was consulted to resolve the conflict. A narrative synthesis of the economic evaluations was conducted to report the study findings [25]. The narrative synthesis focused on important contextual factors of the available literature, including the countries analysed, the target populations represented, types of interventions and technical aspects of the economic analysis including costs, effectiveness and the sources of such data, discounting and perspectives. The country-level data was aggregated regionally and into income brackets based on the 2021 revised categorisations of the World Bank [26]. In addition to these main themes, the researchers also recorded details on first and corresponding authorship and their affiliations, whether any conflicts of interest were declared and what they were as well as funding sources. The extraction tables were generated using Microsoft Excel and data visualisation was conducted using Datawrapper [27].

To understand which interventions were deemed cost-effective and to make international comparisons, all extracted incremental cost-effectiveness ratios (ICERs) that reported a cost per QALY ICERs were converted to international Dollars using purchasing price parity (PPP) indices, consistent with the approach reported by Velasco et al. 2012 [28, 29]. The ICER values were also inflated to the 2020 cost year, where necessary. To attempt to understand whether the transformed ICER values could be considered cost-effective in different countries, the ICERs were presented next to the cost-effectiveness threshold ranges for low/middle income and middle/high income countries, reported by Woods et al. [30]. The country income levels were classified according to the World Bank income classifications, and the values inflated from 2013 to 2020 values to $18–600 for low/middle income countries, and $2,576–10,081 for middle/high income countries.

### Quality assessment

The quality of the data used in the included economic evaluation studies was assessed using an adapted framework for the hierarchy of evidence scoring system detailed in Cooper et al. [31]. Where multiple sources of evidence were used for a single dimension, the highest ranked source of evidence was recorded for consistency.

## Results

**Fig. 1:**
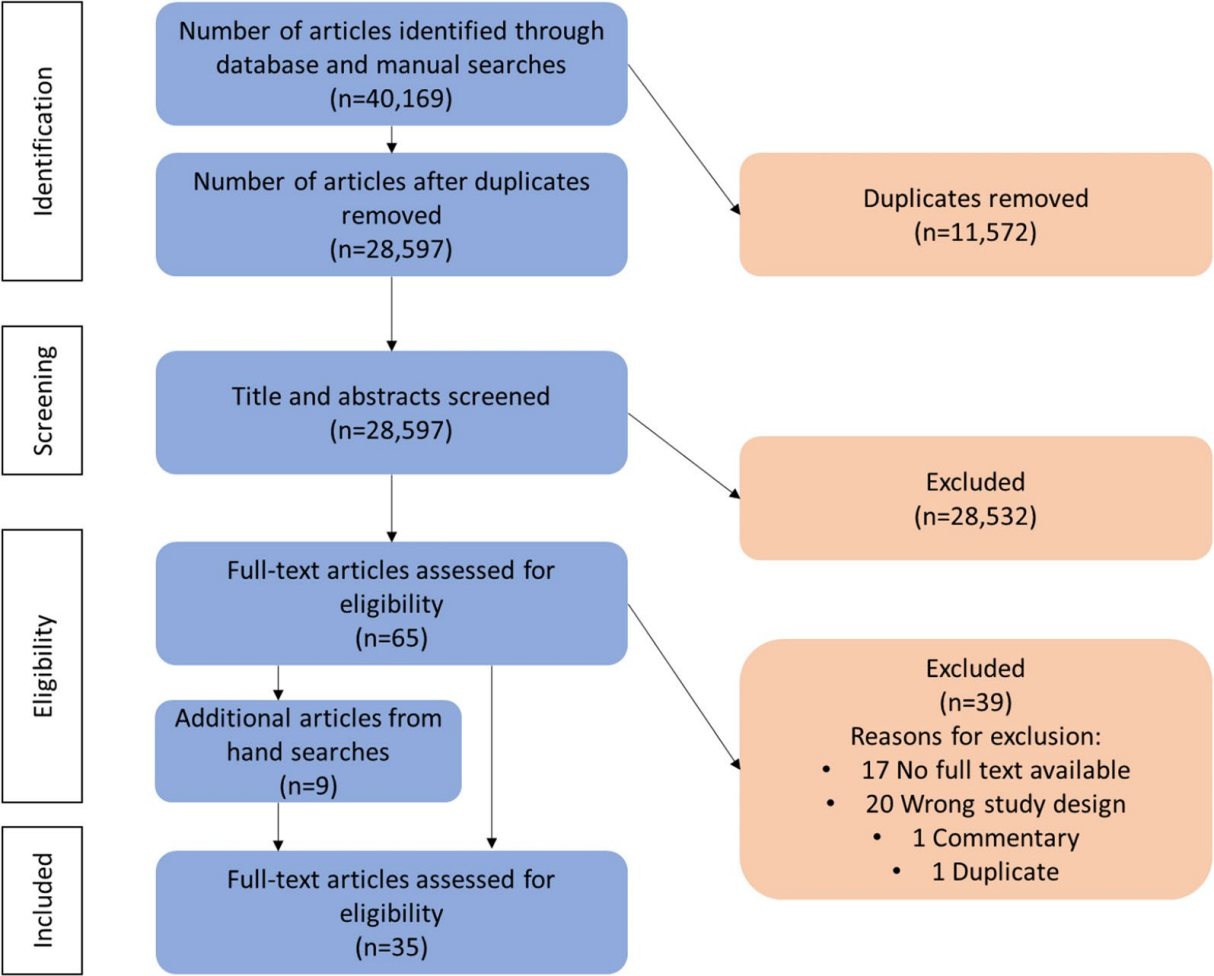
PRISMA Flowchart

The database search yielded 40,169 articles, 11,572 of which were identified as duplicates by Covidence. Out of 28,597 titles and abstracts screened, 65 article full-texts were reviewed and considered for inclusion. Their reference lists were also hand searched for further relevant articles. Articles were most commonly excluded at the full-text stage due to being determined to have the wrong study design, no available full-text, and in one case there was an identified duplicate that had not already been identified by the Covidence software. This resulted in 35 articles that fulfilled the inclusion criteria. The PRISMA flowchart is illustrated in Fig. 1.

### Summary of included studies

Table 1 below summarises the key features of evaluated interventions. All included studies were published between the years of 2001–2021. Health facilities were the most common study setting (n = 26), followed by community (n = 5), and other, which included prisons and multi-sectoral interventions (n = 3). The evaluated interventions included a range of intervention types, such as health care processes and guidelines (n = 20), pharmaceutical intervention (n = 5), antimicrobial stewardship (n = 4), awareness generation activities (n = 2), government policies and legislation (n = 2), medical technologies (n = 1), and combination of pharmaceutical intervention and health care processes and guidelines (n = 1).

**Table 1:** Summary of included studies

Author	Year	Country/territory of analysis	Region	Income level	Setting	Intervention	Perspective
Clancy et al. [32]	2006	US	North America	High	Health facilities	Health care processes and guidelines	Health care system or health care payer or hospital
D'Agata et al. [33]	2018	US	North America	High	Health facilities	Antimicrobial stewardship	Health care system or health care payer or hospital
Fox et al. [34]	2015	US	North America	High	Community	Pharmaceutical intervention	Health care system or health care payer or hospital
Gidengil et al. [35]	2015	US	North America	High	Health facilities	Health care processes and guidelines	Health care system or health care payer or hospital
Gurieva et al. [36]	2013	Netherlands	Europe and Central Asia	High	Health facilities	Health care processes and guidelines	Health care system or health care payer or hospital
Harding-Esch et al. [37]	2020	England	Europe and Central Asia	High	Health facilities	Pharmaceutical intervention	Health care system or health care payer or hospital
Ho et al. [38]	2016	Hong Kong	East Asia and Pacific	High	Health facilities	Health care processes and guidelines	Health care system or health care payer or hospital
Höjgård et al. [39]	2015	Sweden	Europe and Central Asia	High	Other	Health care processes and guidelines	Societal
Hubben et al. [40]	2011	US	North America	High	Health facilities	Health care processes and guidelines	Health care system or health care payer or hospital
Jakab et al. [41]	2015	Multiple (WHO European Region)	Europe and Central Asia	High	Not specified	Government policies and legislations	Health care system or health care payer or hospital
Jansen et al. [42]	2009	Netherlands	Europe and Central Asia	High	Community	Pharmaceutical intervention	Other
Jayaraman et al. [43]	2018	US	North America	High	Health facilities	Health care processes and guidelines	Health care system or health care payer or hospital
Kip et al. [44]	2015	Netherlands	Europe and Central Asia	High	Health facilities	Health care processes and guidelines	Health care system or health care payer or hospital
Meropol et al. [45]	2008	US	North America	High	Community	Health care processes and guidelines	Other
Mewes et al. [46]	2019	US	North America	High	Health facilities	Antimicrobial stewardship	Health care system or health care payer or hospital
Oppong et al. [47]	2013	Norway, Sweden	Europe and Central Asia	High	Health facilities	Medical technologies	Health care system or health care payer or hospital
Oppong et al. [48]	2016	Belgium, France, Germany, Italy, Netherlands, Poland, Slovakia, Slovenia, Spain, Sweden, and UK (England and Wales)	Europe and Central Asia	High	Health facilities	Pharmaceutical intervention	Health care system or health care payer or hospital
Oppong et al. [49]	2018	Belgium, Netherlands, Poland, Spain, UK (England and Wales)	Europe and Central Asia	High	Health facilities	Awareness generation activities	Health care system or health care payer or hospital
Pham et al. [50]	2016	Vietnam	East Asia and Pacific	Lower-middle	Community	Health care processes and guidelines	Health care system or health care payer or hospital
Phillips et al. [51]	2021	South Africa	Sub-Saharan Africa	Upper-middle	Community	Pharmaceutical intervention	Health care system or health care payer or hospital
Puzniak et al. [52]	2004	US	North America	High	Health facilities	Health care processes and guidelines	Health care system or health care payer or hospital
Robotham et al. [53]	2011	England and Wales	Europe and Central Asia	High	Health facilities	Health care processes and guidelines	Health care system or health care payer or hospital
Robotham et al. [54]	2016	UK	Europe and Central Asia	High	Health facilities	Health care processes and guidelines	Health care system or health care payer or hospital
Ruiz-Ramos et al. [55]	2017	Spain	Europe and Central Asia	High	Health facilities	Antimicrobial stewardship	Health care system or health care payer or hospital
Scheetz et al. [56]	2009	US	North America	High	Health facilities	Antimicrobial stewardship	Health care system or health care payer or hospital
Smith et al. [57]	2006	UK	Europe and Central Asia	High	Other	Government policies and legislations	Other
Tran et al. [58]	2016	US	North America	High	Health facilities	Awareness generation activities	Health care system or health care payer or hospital
Voermans et al. [59]	2019	US	North America	High	Health facilities	Antimicrobial stewardship	Health care system or health care payer or hospital and societal
Wang et al. [60]	2020	China	East Asia and Pacific	Upper-middle	Health facilities	Health care processes and guidelines	Health care system or health care payer or hospital
Wassenberg et al. [61]	2010	Netherlands	Europe and Central Asia	High	Health facilities	Health care processes and guidelines	Health care system or health care payer or hospital
Wilton et al. [62]	2001	US and South Africa	Multiple Region	High and upper-middle	Health facilities	Health care processes and guidelines	Health care system or health care payer or hospital
Win et al. [63]	2015	Singapore	East Asia and Pacific	High	Health facilities	Health care processes and guidelines	Health care system or health care payer or hospital
Winetsky et al. [64]	2012	Latvia, Russia and Tajikistan	Europe and Central Asia	High, upper-middle and lower-middle	Other	Health care processes and guidelines	Health care system or health care payer or hospital
You et al. [65]	2012	Hong Kong	East Asia and Pacific	High	Health facilities	Health care processes and guidelines	Health care system or health care payer or hospital
You et al. [66]	2018	Hong Kong	East Asia and Pacific	High	Health facilities	Pharmaceutical intervention and health care processes and guidelines	Health care system or health care payer or hospital

Healthcare processes and guidelines include interventions included screening, surveillance and infection control mechanisms such as isolation, disinfectant application and the use of personal protective equipment. Stewardship programs broadly focused on the rational use of antimicrobials and were tailored to the specific contexts. For instance, Voermans et al. [59] describe an intervention of using procalcitonin algorithm to guide clinical decisions on drug use for lower-respiratory tract infection (LRTI) patients admitted in hospital. Awareness generation or educational interventions have been used in two studies in this review, a dialysis clinic and another was a training initiative for general practitioners in primary care when testing and communications when diagnosing patients presenting with respiratory symptoms [49]. The only medical technology included in this review was a study about a rapid point of care test for improved diagnosis and prescription of antibiotics for those with suspected LRTIs [47]. Of the two studies that analysed government policies or legislation, one analysed the role of macroeconomic approaches such as in tackling AMR while another examined the cost-effectiveness of a comprehensive action plan for multi-drug resistant TB in the WHO European region [41, 57].

Twenty-seven out of 35 studies had received funding support and 26 were authored by academics. Two studies reported first authorship from private consultancies. Sixteen of the 35 included studies did not report conflicts of interest in these studies. Of the 19 that did, only approximately half (n = 9) stated they had no conflicts of interest for the study.

### Objective 1: What interventions to address AMR have been the subject of an economic evaluation?

Given the One Health lens of the review, it was notable that 34 of the 35 focussed on interventions to address AMR exclusively among human populations. Only one study addressed livestock-associated methicillin-resistant *Staphylococcus aureus* (LA MRSA) [39], and was focussed on the transmission of LA-MRSA from animal to human populations. None of the reviewed studies considered AMR concerns in the environment in any capacity.

Twenty out of 35 studies compared types of health care processes and guidelines. These interventions focussed on screening, diagnostics, surveillance and isolation systems in primary clinics, hospitals and tertiary facilities. Only two were community-focussed interventions, that used modified guidelines to ensure greater adherence to prescribed antimicrobial therapy and also as a means to implement a more stringent system of prescription itself [45, 50]. Five studies evaluated different types of ASPs, such as stewardship teams to guide clinical decision-making compared to routine care, or the use of bio-markers for patients with lower-respiratory tract infections. All of the ASPs identified in the review were hospital-based interventions [33, 46, 55, 56, 59].

Five studies detailed pharmaceutical interventions, three in community settings and two in clinics, which analysed recommendations for drug prescription such as amoxicillin for acute LRTIs, preventative fluoroquine therapy for drug-resistant tuberculosis and pre-exposure prophylaxis (PrEP) for patients with HIV. In other cases, these interventions compared two alternate courses of drugs or scenarios where a second- or third-line of drugs may be more cost-effective [34, 37, 42, 48, 51].Three of these interventions were at the community-level and at primary care facilities, including preventive daily drugs against multi-drug resistant tuberculosis. One study in Hong Kong used a combination of interventions; an expanded screening program with pharmaceutical prescriptions for intensive care unit (ICU) patients [66].

Only two studies considered broader national plans as an intervention: One study considered interventions that were specifically related to government policies and legislations that could influence the production, manufacturing and sale of antimicrobials [57], whilst the other focussed on the impact on TB specifically [41]. Examples of these interventions include, regulations, taxes, tariffs and other macroeconomic instruments. Education and training interventions were also being used to address AMR with two studies of this kind included in the review [49, 58]. Only a single study in our review reported on a new, point of care testing method [47]; no other examples of technological solutions for AMR were identified.

Not all included studies focussed on individual pathogens, though the majority did (26/35). The most common pathogen of focus was MRSA, whilst others included minority multi-drug resistant tuberculosis, HIV, gonorrhoea, carbapenem-resistant *Enterobacteriaceae*. Of the nine studies that did not specify an individual pathogen, they either described the class or location of infections, such as acute respiratory or LRTIs, intrabdominal infections, bloodstream infections, sepsis, nosocomial infections and multi-drug resistant organisms [33, 42, 46, 47, 49, 55, 56, 58, 60].

### Objective 2: In what types of setting (by income level and geography) have these economic evaluations been focused?

**Fig. 2:**
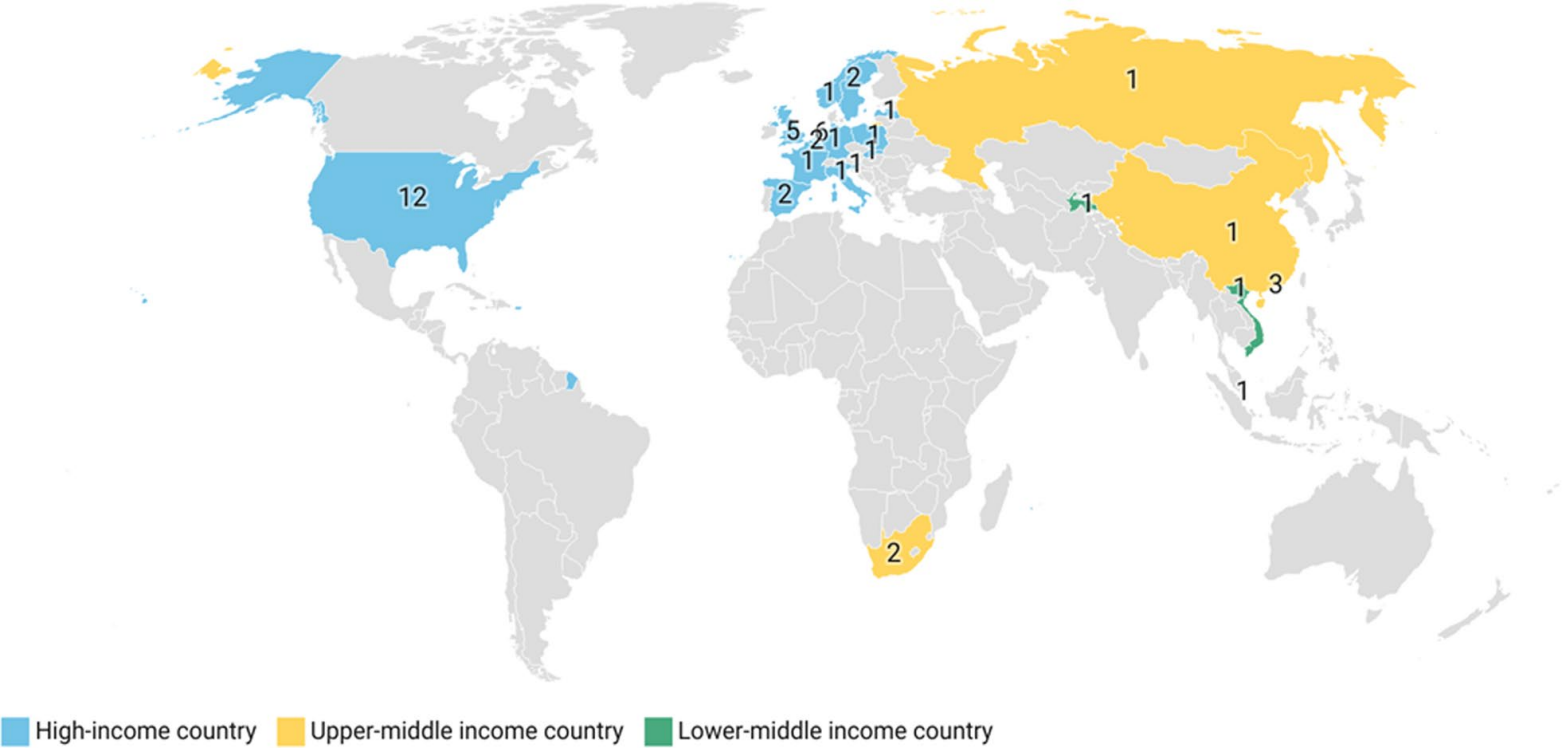
Geographical study populations of included articles in the review by income level. Income and regional categorisations are based on World Bank guidelines. Countries or territories listed: Belgium, China, France, Germany, Hong Kong, Italy, Latvia, Netherlands, Norway, Poland, Russia, Singapore, Slovakia, Slovenia, South Africa, Spain, Sweden, Tajikistan, United Kingdom, United States of America, Vietnam. *One study did not report country level data and has hence not been included in the figure above. **All countries specifically identified in included multi-country studies have been depicted on the map, as such the number of countries represented is greater than the total number of included studies

Twenty-nine studies were conducted in a single country or territory, whereas six studies were conducted in multiple countries or territories. Thirty of the 35 studies were conducted in high-income settings, the majority of which were concentrated in the USA and in Europe; other countries and territories included Hong Kong and Singapore. The upper middle-income settings that were analysed were China and South Africa and Russia, the latter was part of one study that analysed screening for multi-drug resistant tuberculosis for prisoners in the Former Soviet Union (FSU) bloc. The other two countries included in the FSU study were Latvia, a high-income country from Europe and another Tajikistan, a lower-middle income country. The only individual study which focussed on a lower-middle income country in this review was for Vietnam in East Asia. This review has no representation from the South Asian or Latin American and Caribbean region, as well as from low-income countries which includes much of the African continent (see Fig. 2).

### Objective 3: Which interventions have been estimated to be cost-effective, and has this result been replicated in other settings/contexts?

**Fig. 3:**
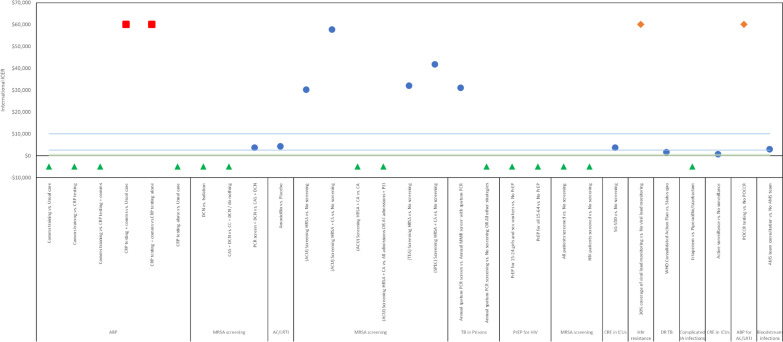
Cost-Effectiveness of Interventions with an Impact on AMR (International Dollar Incremental Cost-Effectiveness Ratio). *ABP* antibiotic prescribing; *AC/LRTI* acute cough or lower respiratory tract infection; *ACU* acute setting; *AMS* Antimicrobial stewardship; *CA* checklist activated; *CAS* chromogenic agar screen; *CRE* carbapenem-resistant enterobacteriaceae; *CRP* C-reactive protein; *DCN* decolonisation; *HIV* human immune-deficiency syndrome; *HRSA* high-risk speciality admissions; *IA* intra-abdominal; *ICER* incremental cost-effectiveness ratio; *ICU* intensive care unit; *MMR* measles, mumps and rubella; *MRSA* methicillin-resistant Staphylococcus aureus; *PCR* polymerase chain reaction; *POCCR* point-of-care C-reactive protein; *PrEP* pre-exposure prophylaxis; *SG-SDD* surveillance-guided selective digestive decontamination; *SPEC* specialist services setting; *TB* tuberculosis; *TEA* teaching hospital setting; *WHO* World Health Organization. Blue dot corresponds to the ICER value on the Y axis. Green triangle means that the intervention (first mentioned technology) dominates the comparator (latter mentioned). Red square means that the intervention (first mentioned technology) was dominated by the comparator (latter mentioned). Orange diamond denotes interventions with Y axis positions adjusted to improve the presentation of the figure, the actual ICER values are listed in the footnotes. Light green lines denote the upper and lower limits of the low/middle-income country cost-effectiveness threshold ($18–600). Blue lines denote the upper and lower limits of the middle/high-income country cost-effectiveness threshold ($2,576–10,081). Adjusted ICER values: 30% coverage of viral load monitoring vs. No viral load monitoring for HIV resistance ($41,476,437/QALY); POCCR testing vs. No POCCR for ABP for AC/LRTI ($106,606)

The subgroup analysis on cost-effectiveness judgements of interventions was limited only to studies which reported a cost per QALY or cost per DALY outcome (n = 14), to allow comparison against explicit cost-effectiveness thresholds. This excluded the majority of the identified studies. A DALY was assumed to be equal to a QALY for this analysis. The results of the subgroup analysis are graphically illustrated in Figs. 3, 2. Due to the small number of studies considered in this subgroup analysis and heterogeneous characteristics of interventions, comparators and study settings, it was not possible to effectively compare, contrast and discuss the consensus of the cost-effectiveness literature for specific interventions.

Three of the 14 studies in this subgroup analysis considered interventions to improve antibiotic prescribing practices, namely ASPs and improved diagnostic methods. Point-of-care C-reactive (POCCR) protein testing was considered in both analyses and was estimated to be cost-effective in the analysis for Belgium, Netherlands, Poland, Spain, England and Wales but not in the analyses for Norway and Spain. The first author was the same for both articles which suggests that the cost-effectiveness conclusion is likely to be highly context-specific.

Screening strategies and treatment strategies for MRSA were analysed in three studies, two in the United Kingdom or England and Wales, and one in Singapore. The studies considering screening strategies seemed to be consistent in that it was more cost-effective to screen in the highest-risk settings (acute care settings/patients with co-morbidities). However, in Singapore, screening all patients was estimated to be dominant compared to no screening whereas in the UK, Robotham et al. 2016 at all found no screening to be cost-effective at the national cost-effectiveness threshold. However, notably this study did not consider the secondary health and cost impacts from the prevention of AMR. Robotham et al. 2011 estimated that that decolonisation was more cost-effective than an isolation for MRSA-positive patients.

Only one of the studies included in this analysis was conducted in an LMIC (Vietnam). However, as the ICERs were converted to an International Dollar scale we can infer which interventions may be cost-effective in LMICs, though locally contextualised analysis should be performed to investigate this further. These included: communications training or C-reactive protein testing to reduce inappropriate antibiotic prescribing; decolonisation of MRSA-infected patients, various screening strategies for MRSA; annual sputum PCR screening for TB in prisons; PrEP for high-risk groups or all adults in high-prevalence settings; surveillance and decontamination strategies for CRE in ICUs; comprehensive national strategies to combat drug-resistant TB; and finally, antimicrobial stewardship team consultations to improve bloodstream infection prescribing.

### Objective 4: What economic evaluation methods or techniques have been used to evaluate these interventions?

As displayed in Table 2, the majority of the economic evaluations were model-based (n = 31), and used a health care system or health care payer or hospital perspective (n = 30) and > 1-year time horizons (n = 12), the maximum of which was fifty years. The most common method employed was cost-utility analysis (n = 14) and other cost-effectiveness analyses (n = 14) and cost-consequence analyses (n = 5). Decision tree, mathematical, and statistical model were the most commonly used types of models, with 14, 6, and 5 studies respectively. There were also 6 studies that used Markov or Markov microsimulation models. Table 3 summarises the methods and techniques used to evaluate the interventions in the included studies.

**Table 2:** Methodological summary of included studies

Author	Year	Method	Type of analysis	Model type	Time horizon
Clancy et al. [24]	2006	Alongside clinical study	CCA	Mathematical model	2 years
D'Agata et al. [25]	2018	Model based	CCA	Decision tree	1 year
Fox et al. [26]	2015	Model based	CCA	Decision tree plus other model	20 years
Gidengil et al. [27]	2015	Model based	CEA	Markov microsimulation	1 year
Gurieva et al. [28]	2013	Model based	CEA	Mathematical model	10 years
Harding-Esch et al. [29]	2020	Model based	CEA	Decision tree	Not specified
Ho et al. [30]	2016	Model based	CUA	Decision tree	< 1 year
Höjgård et al. [31]	2015	Model based	CBA	Decision tree	1 year
Hubben et al. [59]	2011	Model based	CEA	Mathematical model	15 years
Jakab et al. [49]	2015	Model based	CUA	Statistical model	5 years
Jansen et al. [47]	2009	Model based	CUA	Decision tree	5 years
Jayaraman et al. [57]	2018	Model based	CEA	Decision tree	< 1 year
Kip et al. [41]	2015	Model based	CEA	Decision tree	< 1 year
Meropol et al [32]	2008	Model based	CEA	Decision tree	Not specified
Mewes et al. [33]	2019	Model based	CEA	Decision tree	< 1 year
Oppong et al. [34]	2013	Alongside clinical study	CUA	Statistical model	< 1 year
Oppong et al. [35]	2016	Alongside clinical study	CUA	Statistical model	< 1 year
Oppong et al. [36]	2018	Alongside clinical study	CUA	Statistical model	< 1 year
Pham et al. [37]	2016	Model based	CUA	Mathematical model	15 years
Phillips et al. [38]	2021	Model based	CUA	Markov microsimulation	50 years
Puzniak et al. [39]	2004	Model based	CBA	Decision tree	1 year
Robotham et al. [40]	2011	Model based	CUA	Markov microsimulation	5 years
Robotham et al. [42]	2016	Model based	CUA	Markov microsimulation	5 years
Ruiz-Ramos et al. [43]	2017	Model based	CEA	Decision tree plus other model	1 year
Scheetz et al. [44]	2009	Model based	CUA	Decision tree	Not specified
Smith et al. [45]	2006	Model based	Other	Mathematical model	Not specified
Tran et al. [46]	2016	Model based	CCA	Decision tree	1 year
Voermans et al. [48]	2019	Model based	CEA	Decision tree	< 1 year
Wang et al. [50]	2020	Model based	CEA	Decision tree plus other model	1 year
Wassenberg et al. [51]	2010	Model based	CEA	Mathematical model	1 year
Wilton et al. [52]	2001	Model based	CCA	Decision tree plus other model	< 1 year
Win et al. [53]	2015	Model based	CUA	Statistical model	2 years
Winetsky et al. [54]	2012	Model based	CUA	Markov	10 years
You et al. [55]	2012	Model based	CEA	Decision tree	Not specified
You et al. [56]	2018	Model based	CUA	Markov	< 1 year

*CBA* cost–benefit analysis; *CCA* cost-consequence analysis; *CEA* cost-effectiveness analysis; *CUA* cost-utility analysis

**Table 3:** Economic evaluation method, analysis and model used to assess the interventions in studies

No	Intervention	Method	Type of analysis						Type of model structure					
		Alongside clinical study (n = 4)	Model based (n = 31)	CEA (n = 14)	CUA (n = 14)	CBA (n = 1)	CCA (n = 5)	Other (n = 1)	Markov (n = 2)	MMS (n = 4)	DT (n = 14)	DT and other (n = 4)	MM (n = 6)	SM (n = 5)
1	Antimicrobial stewardship	–	4	2	1	–	1	–	–	–	3	1	-	–
2	Awareness generation activities	1	1	–	1	–	1	–	-	–	1	–	–	1
3	Government policies and legislations	–	–	–	1	–	-	1	–	–	–	–	1	1
4	Health care processes and guidelines	1	19	11	6	1	2	–	1	3	8	2	5	1
5	Medical technologies	1	1	–	1	–	–	–	–	–	–	–	–	1
6	Pharmaceutical intervention	1	4	1	3	–	1	–	–	1	2	1	–	1
7	Pharmaceutical intervention and health care processes and guidelines	–	1	–	1	–	–	–	1	–	–	–	–	–

*CBA* cost–benefit analysis; *CCA* cost-consequence analysis; *CEA* cost-effectiveness analysis; *CUA* cost-utility analysis; *DT* decision tree; *MM* mathematical modelling; *MMS* Markov microsimulation; *SM* statistical model

### Objective 5: What kind of data has been used in conducting economic evaluations for these interventions? What is the quality of this data?

**Fig. 4:**
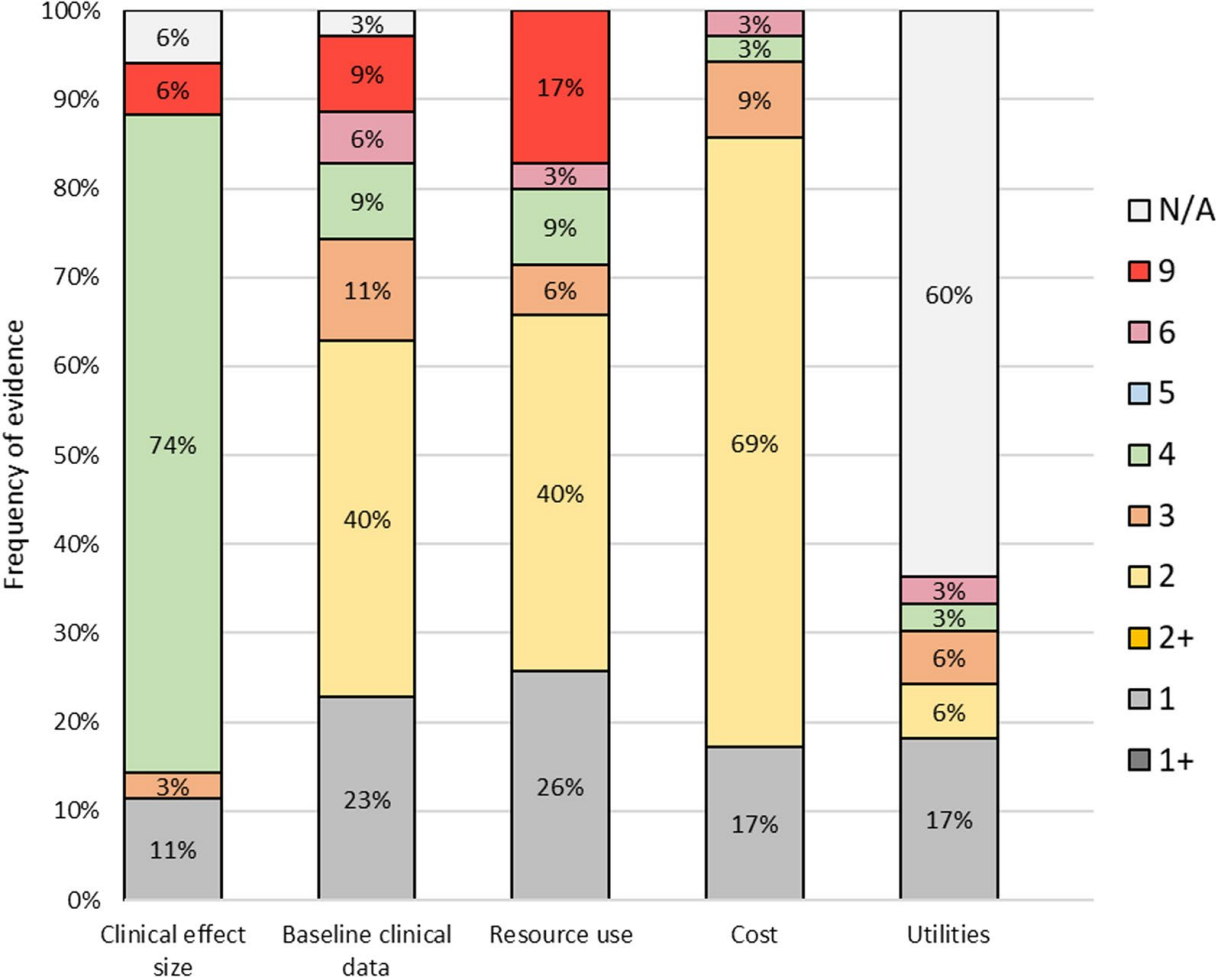
Quality of data sources. N = 35. A score of 1 denotes the highest quality evidence, a score of 6 is the lowest quality evidence, and a score of 9 is when the data source is not stated or unclear. Details of the scoring hierarchies are detailed in the Additional files 1 and 2

Thirty-five studies were assessed for the quality of data sources used in the included studies using the hierarchies proposed by Cooper et al. [31] (Fig. 4). 26 studies (74%) used observational studies for the clinical effect sizes. Meanwhile, the baseline clinical data were predominantly (63%, n = 22) sourced from recent case series or analysis of reliable administrative databases covering patients solely from the jurisdiction of interest specifically conducted for the study. In terms of resource use, 23 (66%) studies used either prospective data collection for the specific study or used published results or reliable administrative data in the same jurisdiction. As for the cost inputs, 24 studies (69%) used reliable databases of the same jurisdiction, published within 5 years of the study’s cost year. Only 40% (n = 14) of the included studies used QALYs as an outcome measure, with six of these studies sourced from high-quality data sources, classified as direct or indirect utility assessment for the specific study using validated tools.

## Conclusions

This manuscript represents an addition to the limited pool of literature on systematic reviews of economic evaluations impacting AMR. It is the first review since 2002 that considers a broad set of interventions, and the first to include non-human health interventions, though only a single study of this type was identified. To meaningfully build upon the limited body of literature that exists in this area and inform future research, the review was designed with a wide scope, in terms of types of interventions, pathogens, study populations. This approach resulted in a large number of abstracts being identified and subsequently reviewed, Specifically, this review, despite its breadth, did not identify any studies from the Latin American, and Caribbean region as well as from a majority of the African countries, although this may be a result of only including English language articles. AMR is growing fastest in LMIC settings, where resources are particularly constrained, and economic evidence could help improve the efficiency response to the threat to AMR in these settings. The increased importance of global health security issues, in the context of COVID-19 and other outbreaks, serves as a reminder of the importance of investment in addressing AMR. We urge local and international researchers and funders to prioritise conducting economic evaluations focussed on reducing AMR in LMICs.

The review highlighted that although there were several economic evaluations of ASPs identified, these studies need to be replicated in other settings, particularly in LMICs as the included studies predominantly focussed on high income settings. This could in part be due to poorer data availability in LMIC countries to populate economic evaluations, which could be abated by increased collection of relevant data in LMICs. Conducting economic evaluations on AMR may also be perceived as a lower research priority in LMIC settings, and due to the complexity of evaluating some interventions comprehensively, technical capacity constraints could have contributed to the lack of research in this area.

The review has demonstrated that there were a large proportion of CEA-but-not-CUA studies (i.e. did not use QALYs or DALYs), this may be due to the difficulty researchers have found in trying to convert certain outcomes (e.g. reduced antimicrobial consumption or infections avoided) into generic quantifiable health outcomes such as QALYs. Using QALYs and DALYs in cost-effectiveness analyses is preferable as these can then be compared to existing and accepted cost-effectiveness thresholds in order to inform policy decisions and improve allocative efficiency. Alternatively, there could be distinct and accepted cost-effectiveness thresholds specific to AMR using other measures than cost per QALY (e.g. antibiotic doses avoided) in order for CEA-but-not-CUA studies to have a greater impact on policy.

Our quality of evidence assessment highlighted that researchers in many cases have not used, presumably due to lack of availability, high quality data for their AMR studies. This is particularly true for the impact or efficacy of the interventions themselves, which most often used non-analytic study evidence. Furthermore, our quality of evidence section overstates the average quality of evidence used, as in stations where multiple input sources were used for a single dimension (which is often the case in economic evaluations) the highest quality of evidence score was recorded in order to be consistent. This review highlighted that the One Health approach to AMR has not yet been reflected in the economic evidence; only one identified study was not focussed on human health [39], though this could be due to flaws in our search terms in identifying evidence of this type.

As stated in the methods, it was difficult to identify interventions that affect AMR, as this could be the case for a huge number of interventions and the authors may or may not have chosen to consider that facet of the intervention. Therefore, in order to have a consistent and explicit approach in the screening process, only abstracts which stated an impact on AMR, in terms of antimicrobial use or resistance were included. As such, there may have been articles that were missed which only stated an impact in the full text, however this step was necessary in order to keep the number of articles included at the full-text screening stage to a feasible amount. The inclusion criteria could be improved upon in future research. Only English language studies were considered as the research team did not have the capacity to review articles in other languages.

Only a limited review of the methodologies of the economic evaluations was performed for the purposes of this study. However one methodological recommendation is that research could make better use of recent efforts to estimate the secondary economic costs of antibiotic consumption (e.g. by Shrestha et al. [67]), which account for how changes in consumption impact the speed and therefore costs of AMR, and incorporate these into future economic analyses. There would be substantial value in a more in-depth review of the methods of economic evaluations relevant to AMR. A review of this type could also identify best practices and technical recommendations on how these studies could be improved to ensure they reflect the nuances of AMR and comprehensively reflect the costs and health outcome impacts of AMR.

A brief review of the literature of clinical trials conducted for interventions to reduce AMR highlighted numerous interventions that have not yet been the subject of an economic evaluation [68–72]. These included educational and behaviour change interventions to improve prescribing, or to educate patients on their treatment seeking and adherence behaviour, and even interventions to reduce antibiotic use in animals. Future research in this area should focus on economic evaluations of interventions to combat AMR that have not previously been the subject of an economic evaluation, which would be useful for broadening the literature base and facilitating future research. The most similar previous systematic review on this topic by Wilton et al. 2001 also found that most of the included studies were conducted for high-income settings, for hospital-based settings and interventions. Wilton et al. described a “paucity of evidence [which] makes definitive recommendations concerning which strategies should be pursued, when, where and how, impossible”. This review has demonstrated that this statement largely remains to be true 20 years later.

### Supplementary Information

**Additional file 1**. Cooper et al. Hierarchy of Evidence Ranking Scores.

**Additional file 2**. Database search terms.

## Data Availability

Cooper et al. Hierarchy of Evidence Ranking Scores. Database search terms.
