# Correction: A systematic literature review of economic evaluation studies of interventions impacting antimicrobial resistance

**DOI:** 10.1186/s13756-023-01314-z

**Published:** 2023-09-29

**Authors:** Chris Painter, Dian Faradiba, Kinanti Khansa Chavarina, Ella Nanda Sari, Yot Teerawattananon, Kristina Aluzaite, Aparna Ananthakrishnan

**Affiliations:** 1 Health Intervention and Technology Assessment Program (HITAP), Ministry of Public Health, Nonthaburi, Thailand; 2 Mahidol-Oxford Tropical Medicine Research Unit, Bangkok, Thailand; 3 Centre for Tropical Medicine and Global Health, Nuffield Department of Medicine, University of Oxford, Oxford, UK; 4 National University of Singapore, Singapore, Singapore; 5 Centre for Health Economics, University of York, York, UK

**Antimicrobial Resistance & Infection Control (2023) 12:69**

10.1186/s13756-023-01265-5

The original article [1] contains an error in Affiliation #2. The correct affiliation can be viewed in this Correction article and is as follows:

‘Mahidol-Oxford Tropical Medicine Research Unit, Bangkok, Thailand.’

