# Supplementary material for: A systematic literature review of economic evaluation studies of interventions impacting antimicrobial resistance
Source: Antimicrob Resist Infect Control. 2023 Jul 13;12:69. doi: 10.1186/s13756-023-01265-5 (PMC10339577; doi:10.1186/s13756-023-01265-5)
Supplement: Supplementary file 1 — Additional file 1. Cooper et al. Hierarchy of Evidence Ranking Scores. [file 13756_2023_1265_MOESM1_ESM.docx]

# Supplementary Materials

## Cooper et al. Hierarchy of Evidence Ranking Scores

| **Rank** | **Data components** |
| --- | --- |
| ***Clinical effect sizes, adverse events & complications*** | |
| 1+ | Meta-analysis of RCTs with direct comparison between comparator therapies, measuring final outcomes |
| 1 | Single RCT with direct comparison between comparator therapies, measuring final outcomes |
| 2+ | Meta-analysis of RCTs with direct comparison between comparator therapies, measuring surrogate outcomes  Meta-analysis of placebo-controlled RCTs with similar trial populations, measuring the final outcomes for each individual therapy |
| 2 | Single RCT with a direct comparison between comparator therapies, measuring surrogate outcomes  Single placebo-controlled RCT with similar trial populations, measuring the final outcomes for each individual therapy |
| 3+ | Meta-analysis of placebo-controlled RCTs with similar trial populations, measuring the surrogate outcomes |
| 3 | Single placebo-controlled RCTs with similar trial populations, measuring the surrogate outcomes for each individual therapy |
| 4 | Observational studies (e.g., case control) |
| 5 | Non-analytic studies (e.g., case reports) |
| 6 | Expert opinion |
| 9 | Not stated |
| ***Baseline clinical data*** | |
| 1 | Case series or analysis of reliable administrative databases specifically conducted for the study covering patients solely from the jurisdiction of interest |
| 2 | Recent case series or analysis of reliable administrative databases covering patients solely from the jurisdiction of interest |
| 3 | Recent case series or analysis of reliable administrative databases covering patients solely from another jurisdiction |
| 4 | Old case series or analysis of reliable administrative databases; estimate from RCTs |
| 5 | Estimates from previously published economic analyses: unsourced |
| 6 | Expert opinion |
| 9 | Not stated |
| ***Resource use*** | |
| 1 | Prospective data collection or analysis of reliable administrative data for specific study |
| 2 | Recently published results of prospective data collection or recent analysis of reliable administrative data – same jurisdiction |
| 3 | Unsourced data from previous economic evaluations – same jurisdiction |
| 4 | Recently published results of prospective data collection or recent analysis of reliable administrative data – different jurisdiction |
| 5 | Unsourced data from previous economic evaluations – different jurisdiction |
| 6 | Expert opinion |
| 9 | Not stated |
| ***Costs*** | |
| 1 | Cost calculations based on reliable databases or data sources conducted for specific study – same jurisdiction |
| 2 | Recently published cost calculations based on reliable databases or data course – same jurisdiction |
| 3 | Unsourced data from previous economic evaluation – same jurisdiction |
| 4 | Recently published cost calculations based on reliable databases or data course – different jurisdiction |
| 5 | Unsourced data from previous economic evaluation – different jurisdiction |
| 6 | Expert opinion |
| 9 | Not stated |
| ***Utilities*** | |
| 1 | Direct utility assessment for the specific study from a sample either: (a) of the general population, (b) with knowledge of the disease(s) of interest, (c) of patients with the disease(s) of interest  Indirect utility assessment for the specific study from patient sample with disease(s) of interest, using a tool validated for the patient population |
| 2 | Indirect utility assessment for the specific study from patient sample with disease(s) of interest, using a tool not validated for the patient population |
| 3 | Direct utility assessment from a previous study from a sample either: (a) of the general population, (b) with knowledge of the disease(s) of interest, (c) of patients with the disease(s) of interest |
| 4 | Indirect utility assessment from a previous study from patient sample with disease(s) of interest: using tool not validated for the patient population.  Unsourced utility data from previous study – method of elicitation unknown |
| 5 | Patient preference values obtained from a visual analogue scale |
| 6 | Delphi panels, expert opinion |
| 9 | Not stated |
