# Supplementary material for: A systematic literature review of economic evaluation studies of interventions impacting antimicrobial resistance
Source: Antimicrob Resist Infect Control. 2023 Jul 13;12:69. doi: 10.1186/s13756-023-01265-5 (PMC10339577; doi:10.1186/s13756-023-01265-5)
Supplement: Supplementary file 2 — Additional file 2. Database search terms. [file 13756_2023_1265_MOESM2_ESM.docx]

# Supplementary Materials

## EMBASE Search terms

|  | 'economic development'/exp OR 'economic development' OR 'economic model'/exp OR 'economic model' OR 'economic aspect'/exp OR 'economic aspect' OR 'economic recession'/exp OR 'economic recession' OR 'fee'/exp OR 'fee' OR 'budget'/exp OR 'budget' |
| --- | --- |
|  | 'cost'/exp OR 'cost benefit analysis'/exp |
|  | economic*:ti,ab,de,tn OR pharmacoeconomic*:ti,ab,de,tn OR 'economic analysis':ti,ab,de,tn OR 'economic evaluation':ti,ab,de,tn OR 'economic stud*':ti,ab,de,tn OR 'economic modelling':ti,ab,de,tn OR price*:ti,ab,de,tn OR pricing:ti,ab,de,tn |
|  | ("cost benefit" OR "cost effective" OR "cost analysis" OR "cost minimisation" OR "cost utility analysis" OR "budget impact"):ti,ab,de,tn |
|  | (cost OR "cost description" OR "cost saving" OR "cost shar*" OR "cost allocation"):ti,ab,de,tn |
|  | (health*care NEXT/1 cost*):ti,ab,de,tn |
|  | ((cost NEXT/1 estimate*) OR (cost NEXT/1 variable*) OR (unit NEXT/1 cost*)):ti,ab,de,tn |
|  | **(#1 or #2 or #3 or #4 or #5 or #6 or #7)** |
|  | 'antibiotic resistance'/exp OR 'antiviral resistance'/exp OR 'drug resistance'/exp OR 'antifungal resistance'/exp |
|  | ("antimicrobial drug resistan*" OR "drug resistan*" OR "antibiotic resistan*" OR "antimicrobial resistan*" OR "multi-drug resistan*"):ti,ab,de,tn |
|  | ("antimicrobial drug resistan*" OR "antibiotic resistan*" OR "antimicrobial resistan*"):ti,ab |
|  | **(#9 or #10 or #11)** |
|  | **#8 AND #12 AND [english]/lim AND [2000-2021]/py AND [embase]/lim** |

## MEDLINE Search Terms

|  | exp Economic Development/ or exp Models, Economic/ or exp Economic Competition/ or exp Inflation, Economic/ or exp Economic Recession/ or exp "fees and charges"/ or exp Budgets/ |
| --- | --- |
|  | exp "costs and cost analysis" |
|  | (economic* or pharmacoeconomic* or "economic analysis" or "economic evaluation" or "economic stud*" or "economic modelling" or price* or pricing).mp. |
|  | ("cost benefit" or "cost effective" or "cost analysis" or "cost minimisation" or "cost utility analysis" or "budget impact").mp. |
|  | (cost or "cost description" or "cost saving" or "cost shar*" or "cost allocation").mp. |
|  | (health?care adj cost$).mp. |
|  | (cost adj estimate*) or ((cost adj variable*) or (unit adj cost*).mp. |
|  | **(1 or 2 or 3 or 4 or 5 or 6 or 7)** |
|  | exp "Drug Resistance, Microbial"/ or exp "Antibiotic Resistance"/ or exp "Antibiotic Resistance, Microbial"/ or exp "Antimicrobial Drug Resistance" |
|  | ("antimicrobial drug resistan*" or "drug resistan*" or "antibiotic resistan*" or "antimicrobial resistan*" or "multi-drug resistan*").mp |
|  | ("antimicrobial drug resistan*" or "antibiotic resistan*" or "antimicrobial resistan*").tw |
|  | **(9 or 10 or 11)** |
|  | **(8 and 12)** |
|  | **limit 13 to yr="2000 -Current"** |
|  | **limit 14 to english language** |

## Web of Science Search Terms

|  | TS=("Economic Development" OR "Models, Economic" OR "Economic Competition" OR "Inflation, Economic" OR "Economic Recession" OR "fees and charges" OR Budgets) |
| --- | --- |
|  | TS="costs and cost analysis") |
|  | TS=(economic* or pharmacoeconomic* or "economic analysis" or "economic evaluation" or "economic stud*" or "economic modelling" or price* or pricing) |
|  | TS=("cost benefit" or "cost effective" or "cost analysis" or "cost minimisation" or "cost utility analysis" or "budget impact") |
|  | TS=(cost or "cost description" or "cost saving" or "cost shar*" or "cost allocation") |
|  | TS=(health*care cost*) |
|  | TS=((cost estimate*) or (cost variable*) or (unit cost*)) |
|  | **(1 or 2 or 3 or 4 or 5 or 6 or 7)** |
|  | TS=("Drug Resistance, Microbial" OR "Antibiotic Resistance" OR "Antibiotic Resistance, Microbial" OR "Antimicrobial Drug Resistance") |
|  | TS=("antimicrobial drug resistan*" or "drug resistan*" or "antibiotic resistan*" or "antimicrobial resistan*" or "multi-drug resistan*") |
|  | TS=("antimicrobial drug resistan*" or "antibiotic resistan*" or "antimicrobial resistan*") |
|  | **(9 or 10 or 11)** |
|  | **(8 and 12)** |
|  | **Limits: Year: “2000 -Current" and English language only** |
